# Dual-Time-Point FDG Uptake Correlates with Prognostic Factors of Invasive Breast Cancer: Clinical Usefulness of Early Delayed Scanning

**DOI:** 10.3390/diagnostics9020040

**Published:** 2019-04-09

**Authors:** Ji Young Lee, Hee-Sung Song, Jae Hyuck Choi, Chang Lim Hyun

**Affiliations:** 1 Department of Nuclear Medicine, Jeju National University Hospital, Jeju National University School of Medicine, Jeju-si 63241, Korea; easy02000@naver.com; 2 Department of Surgery, Jeju National University Hospital, Jeju National University School of Medicine, Jeju-si 63241, Korea; basson@hanmail.net; 3 Department of Pathology, Jeju National University Hospital, Jeju National University School of Medicine, Jeju-si 63241, Korea; venisua@jejunu.ac.kr

**Keywords:** fluorodeoxyglucose, positron emission tomography/computed tomography, breast cancer, modified dual-phase, retention index

## Abstract

This study investigated correlations between fluorodeoxyglucose (FDG) uptake in tumors as assessed by modified dual-time-point (mDTP) FDG positron emission tomography/computed tomography (PET/CT) in invasive breast cancer (iBC) and several prognostic parameters. Thirty-two women who underwent mastectomies for iBC were retrospectively evaluated. mDTP scanning was performed using standard FDG PET/CT (PET1), followed by early delayed acquisition (PET2) without repositioning and additional CT scanning. Using maximal standardized uptake values on PET1 (SUV1) and PET2 (SUV2) in the primary breast tumor, the percentage changes between SUV1 and SUV2 (retention index, (RI)) were calculated. Prognostic parameters (e.g., tumor size and stage; number of metastatic lymph nodes; histologic grade; expression of estrogen receptor (ER), progesterone, epidermal growth factor receptor (HER-2), and p53; and the Ki-67 labeling index (LI)) were evaluated from the surgical specimens. PET2 scans were conducted 17.7 ± 1.5 min after PET1. RI values gradually increased as the histologic grade increased (*p* = 0.016), and were significantly higher when ER expression was absent (*p* = 0.023) and Ki-67 LI was high (*p* < 0.001). RI values also showed a moderately positive correlation with Ki-67 LI (*r* = 0.629; *p* < 0.001). RI correlated with well-known biologic prognostic factors of iBC and mDTP scanning, which could be used as a substitute for conventional DTP PET.

## 1. Introduction

Breast cancer is the second-most common malignancy in South Korean women. The prediction of breast cancer prognosis is important in determining treatments for affected patients. Many histopathological and immunohistochemical biomarkers are well-established as prognostic factors, including the histologic grade, the Ki-67 labeling index (LI), and the expression of multiple cellular markers: The estrogen receptor (ER), the progesterone receptor (PR), and the epidermal growth factor receptor (HER-2) [1,2,3,4,5,6,7]. These parameters are indicators of tumor growth, invasion, and metastatic potential; notably, most require invasive procedures, such as mastectomy.

F-18 fluorodeoxyglucose (F-18 FDG) positron emission tomography/computed tomography (PET/CT) is commonly used for the diagnosis, staging, and assessment of treatment response in various cancers, given that most malignant tumors are F-18 FDG-avid with an increased glucose metabolism [8,9,10]. A dual-time-point (DTP) F-18 FDG PET was initially developed to improve the accuracy of F-18 FDG PET for differentiating between benign and malignant lesions [11,12,13,14]. Notably, there was a continuous increase in the F-18 FDG uptake in malignant lesions over time; by contrast, the uptake decreases or remains stable after reaching a maximum within 30 min of FDG administration in benign lesions.

Many studies have attempted to elucidate the relationships between biomarkers and metabolic parameters by using DTP PET; they have demonstrated that the retention index (RI), as determined by DTP F-18 FDG PET/CT, correlates significantly with prognostic factors [15,16,17]. However, DTP PET has disadvantages that include delayed scanning time (approximately 120–180 min after F-18 FDG injection) and additional radiation exposure to the patient due to the second CT needed for attenuation correction. A recent study proposed a modified dual-time-point (mDTP) F-18 FDG PET/CT, so-called early delayed scanning, with a shortened scanning time and without additional radiation exposure [18]. 

To the best of our knowledge, no studies have evaluated correlations between mDTP PET parameters of primary breast tumors and any known clinical, pathological, or biological markers. The purpose of this study was to investigate correlations between RI on tumors evaluated by mDTP F-18 FDG PET/CT and well-known prognostic parameters in patients with invasive breast cancer.

## 2. Materials and Methods

### 2.1. Patients

After this study was approved by the relevant institutional review board (IRB No. JEJUNUH 2017-09-001-001), the medical records of 62 patients with histopathologically confirmed invasive breast cancer were retrospectively reviewed. All included patients underwent preoperative F-18 FDG PET/CT using mDTP scanning at our institution between March 2015 and March 2016. A total of 32 women who had undergone mDTP F-18 FDG PET/CT scans and mastectomies without neoadjuvant therapy, in accordance with our institutional guidelines, were finally enrolled. Tumor staging was based on the seventh edition of the American Joint Committee on Cancer [19].

### 2.2. mDTP FDG PET/CT Imaging

F-18 FDG PET/CT was performed using a PET/CT scanner (Biograph mCT, Siemens, Munich, Germany). All patients had >6 h of fasting time and their blood glucose levels were <120 mg/mL prior to the F-18 FDG injection. After the non-contrast CT scan (100 KeV, 40 mAs) was performed, emission images (90 s/bed) were obtained after an intravenous administration of 370 MBq (10 mCi) F-18 FDG from the basal skull to the mid-thigh. All patients underwent standard F-18 FDG PET/CT (PET1), which was acquired approximately 60 min after the FDG injection. This was followed by early delayed FDG PET scanning at 100 min after the injection, without repositioning or additional CT (PET2). Attenuation-corrected PET images (corrected using CT data) were reconstructed on a 512 × 512 matrix using an iterative ordered-subsets expectation maximization algorithm (21 subsets, 2 iterations). CT and F-18 FDG PET scan data were accurately co-registered using commercial software (Singo, TrueD VE31A, Siemens).

PET images were reviewed by experienced nuclear medicine physicians on a dedicated workstation (TrueD). Maximal standardized uptake values (SUV) on PET1 (SUV1) and PET2 (SUV2) were evaluated, and the percentage changes between SUV1 and SUV2 (RI) were calculated by subtracting SUV1 from SUV2 and dividing by SUV1, as follows: Percentage change (%Δ) = (SUV2 − SUV1)/SUV1 × 100. 

### 2.3. Assessment of Prognostic Factors

Clinical characteristics were obtained from patients’ medical records, and breast cancer histology data were acquired from surgical pathology reports. Pathologic features (tumor size, number of metastatic lymph nodes, and tumor; node; metastasis (TNM) stage) and biologic prognostic parameters (histologic grade; tubular differentiation; nuclear pleomorphism; mitotic count; expression levels of ER, PR, HER-2, and p53; and Ki-67 LI) were assessed from primary tumors. Tumor histology and metastatic lymph nodes were identified using tumor slides with hematoxylin and eosin staining. Immunohistochemical studies were performed on paraffin-embedded tumor tissue using primary antibodies for ER, PR, HER-2, p53, and Ki-67.

### 2.4. Statistical Analysis

The Mann–Whitney U test was used to compare PET parameters and categorical prognostic factors, such as the presence of ER, PR, HER-2, p53 expression, and lymph node involvement, as well as Ki-67 LI and tumor size, because these variables showed significant deviations from the normal distribution. The cut-off values of tumor size and Ki-67 LI were determined in the form of calculated median values. The Kruskal–Wallis test was used to evaluate relationships between PET parameters and 3 histopathological subtypes (tubular differentiation, nuclear pleomorphism, and mitotic count). Spearman’s rank correlation analyses were performed between F-18 FDG PET parameters and prognostic biomarkers for continuous variables, such as Ki-67 LI, tumor size, and the number of metastatic lymph nodes. Commercial software (PASW Statistics 18, SPSS Inc., Chicago, IL, USA) was used throughout, and *p* values of 0.05 were considered to be statistically significant.

## 3. Results

### 3.1. Patient Characteristics

Patient characteristics are shown in Table 1. Respective mean values and standard deviation ranges of SUV1, SUV2, and RI were 6.42 ± 5.17, 6.89 ± 5.60, and 0.05 ± 0.10. PET2 scans were conducted 17.7 ± 1.5 min after PET1 without repositioning or additional CT scanning.

### 3.2. Comparisons between Biological Factors and PET Parameters

#### 3.2.1. Comparisons between Biological Factors and SUV1/SUV2 Values

Table 2 shows relationships between metabolic and biological parameters. Tumor size, histologic grade (tubular differentiation, nuclear pleomorphism, and mitotic count), ER status, and Ki-67 were significantly related to SUV1 and SUV2 values. Lymph node involvement, pathologic stage, PR status, HER-2 status, and p53 status were poorly correlated with SUV1 and SUV2.

#### 3.2.2. Comparisons between Biological Factors and RI Values

RI values gradually increased as the histologic grade increased (1–3, *p* = 0.016). RI values of ER-negative tumors (*p* = 0.023) and high Ki-67 LI (*p* < 0.001) were significantly higher than those of ER-positive tumors and low Ki-67 LI. Similar to the results of SUV1 and SUV2 values, lymph node involvement, pathologic stage, PR status, HER-2 status, and p53 status were poorly correlated with RI values.

### 3.3. Correlation Analyses

#### 3.3.1. Ki-67

Table 3 shows correlations between metabolic parameters and histopathologic variables. SUV1, SUV2, and RI values were moderately positively correlated with Ki-67 LI (*r* = 0.706; *p* < 0.001, *r* = 0.744; *p* < 0.001, *r* = 0.629; *p* < 0.001). 

#### 3.3.2. Tumor Size

Moderately positive correlations were detected between tumor size and SUV1, as well as between tumor size and SUV2 (*r* = 0.611; *p* < 0.001, *r* = 0.592; *p* < 0.001); however, tumor size was not significantly correlated with RI values (*p* = 0.07).

#### 3.3.3. Number of Metastatic Lymph Nodes

No significant correlations were found between the number of metastatic lymph nodes and PET parameters.

## 4. Discussion

In this study, we investigated correlations between metabolic parameters of mDTP F-18 FDG PET/CT and multiple clinical, pathological, and biological markers in subjects with invasive breast cancer. Our data showed significant correlations between metabolic parameters and each of the following: Histologic grade, Ki-67 LI, and ER expression. SUV1 and SUV2 were also moderately correlated with tumor size.

Currently, several prognostic biomarkers of breast cancer (e.g., hormone receptor status, HER-2 status, Ki-67 LI, p53 expression, and histologic grade) are considered to be sources of valuable information regarding tumor aggressiveness, as well as the likelihood of response to therapy. Among these prognostic factors, the expression of hormone receptor is particularly useful for guiding treatment decisions in clinical oncology; the presence of Ki-67 LI, a molecular marker of tumor proliferation, has been linked to a poorer prognosis in breast cancer [2,3,4,5,6,7,15]. Several studies have shown that the F-18 FDG uptake in primary breast tumor is positively associated with aggressive tendencies of biologic features [20,21,22,23,24,25]. In our study, higher SUV1 was positively associated with tumor size, histologic grade, ER negativity, and Ki-67 LI, indicating malignant tendency. 

Over the last 10 years, there have been many reports that DTP F-18 FDG PET can be useful for discriminating between benign and malignant lesions, as well as to evaluate equivocal metastatic lesions [26]. These studies were based on the results of previous reports that FDG uptake continues to increase in malignant tumors for several hours after injection, while the uptake in benign lesions decreases or remains stable over time. However, a conventional DTP F-18 FDG PET/CT approach, which requires delayed acquisition at 120–180 min after FDG injection, involves a degree of inconvenience. Additional time is needed to obtain the delayed test (approximately 1–2 h) and additional radiation exposure occurs during the acquisition of delayed images by CT for attenuation correction. An early delayed DTP F-18 FDG PET/CT has been proposed to overcome these problems. mDTP F-18 FDG PET/CT enables lower radiation exposure to patients and shortens the scanning time compared to conventional DTP F-18 FDG PET/CT. Kanae et al. reported that the physiologic radioactivity of bowel secretion can be excluded by the interval time difference between the initial image and the delayed image using mDTP F-18 FDG PET/CT scan [18].

Some studies have investigated whether DTP F-18 FDG PET is related to histopathological and immunohistochemical parameters; most reported studies have used the conventional DTP F-18 FDG PET/CT scan method [15,16,17,27]. Moon et al. suggested that higher RI may be well-correlated with lower ER expression and higher HER-2 expression [15]. In the present study, we found that RI was related to histologic grade, ER negativity, and Ki-67 LI when using the mDTP PET/CT scan. Concordant with our results, Gracia Vicente et al. found that RI was associated with histological grade and Ki-67 LI, as well as ER status, using the conventional DTP FDG PET/CT scan; however, they also reported that RI was correlated with PR and HER-2 statuses, which is not consistent with our findings [16,17]. By contrast, Ozen et al. reported that only PR status was correlated with RI value [27]. These discordant results might be due to the small numbers of subjects in the present study and in previous studies, as well as differences in DTP scan time. Notably, these studies showed that metabolic parameters were correlated with several biological parameters in breast cancer, although some parameters have shown inconsistent results. Importantly, FDG uptake continued to increase for 1–2 h after FDG injection in malignant tumors; as this finding is associated with poor prognosis, it was possible to obtain a correlation between prognostic variables and RI, including an early delayed scan time. To the best of our knowledge, this is the first study to show that RI on mDTP F-18 FDG PET/CT was a prognostic factor in patients with breast cancer. Thus, mDTP F-18 FDG PET/CT may constitute a convenient and noninvasive tool to predict the baseline risk in patients with breast cancer, and is expected to be useful for other FDG-avid solid tumors; this may aid in risk stratification and therapeutic planning in future studies.

The limitations of this study include the small number of patients and its retrospective nature, such that only patients with pathologically proven breast cancer were enrolled. Thus, a selection bias might be present. Second, we could not analyze relationships between metabolic parameters and the molecular type of breast cancer because of the small number of patients. Finally, this study was localized to a single hospital. Therefore, multicenter and prospective studies using larger sample sizes are needed to confirm the suitability of mDTP F-18 FDG PET/CT for the prediction of biological characteristics. 

## 5. Conclusions

This study demonstrated that RI correlated with well-known biologic prognostic factors of invasive breast cancer. Early delayed scanning could substitute for a conventional dual-time-point FDG PET/CT while providing advantages, such as low radiation exposure to patients and a shortened scanning time.

## Figures and Tables

**Table 1:** Characteristics of patients.

**Total Patients, *n***	32
Age, median (interquartile range), years	48 (44–58)
BMI, median (interquartile range), kg/m^2^	24.5 (22.4–27.2)
Lymph node involvement, n (%)	21 (65.6)
Histology type (%)	
Invasive ductal carcinoma	30 (93.8)
Invasive lobular carcinoma	2 (6.2)
Tumor size, median (interquartile range), mm	2.1 (1.6–3.0)
T stage (%)	
T1	16 (50.0)
T2	14 (43.8)
T3	2 (6.2)
Clinical stage	
I	12 (37.5)
II	17 (53.1)
III	3 (9.4)

**Table 2:** Comparisons of metabolic parameters and histopathologic variables.

Histopathologic Variables (*n*)	Mean SUV1 ± SD	*p* Value	Mean SUV2 ± SD	*p* Value	Mean RI ± SD	*p* Value
Tumor size		0.005		0.009		0.309
<2.1 cm (16)	4.1 ± 2.8		4.4 ± 3.2		2.8 ± 10.2	
≥2.1 cm (16)	8.8 ± 6.0		9.4 ± 6.3		6.4 ± 8.7	
Lymph node involvement		0.293		0.242		0.463
No (21)	5.5 ± 4.1		5.9 ± 4.7		3.7 ± 10.6	
Yes (11)	8.2 ± 6.6		8.8 ± 6.9		6.4 ± 7.0	
Pathologic stage		0.103		0.147		0.742
I (12)	4.1 ± 2.8		4.4 ± 3.2		3.0 ± 11.1	
II (17)	7.1 ± 4.7		7.7 ± 5.4		5.7 ± 9.3	
III (3)	11.9 ± 10.5		12.3 ± 10.7		5.0 ± 2.4	
Histologic grade		<0.001		0.001		0.016
1 (9)	2.6 ± 2.2		2.6 ± 2.3		−2.3 ± 9.6	
2 (13)	5.3 ± 2.59		5.6 ± 2.8		4.9 ± 7.9	
3 (10)	11.3 ± 6.0		12.4 ± 6.2		10.5 ± 7.7	
Tubular differentiation		0.003		0.005		0.076
1 (2)	1.0 ± 0.3		0.9 ± 0.2		−11.4 ± 4.2	
2 (10)	3.9 ± 2.5		4.2 ± 2.8		3.9 ± 9.8	
3 (20)	8.2 ± 5.6		8.8 ± 6.0		6.5 ± 8.4	
Nuclear pleomorphism		0.003		0.002		0.002
1 (6)	2.3 ± 1.9		2.2 ± 2.0		−7.4 ± 6.6	
2 (18)	6.2 ± 5.2		6.6 ± 5.3		5.8 ± 7.4	
3 (8)	10.1 ± 4.6		11.2 ± 5.1		10.8 ± 8.0	
Mitotic count		0.001		<0.001		0.01
1 (15)	3.5 ± 2.6		3.6 ± 2.8		−0.2 ± 8.8	
2 (9)	6.0 ± 2.3		6.4 ± 2.5		5.8 ± 8.5	
3 (8)	12.3 ± 6.3		13.6 ± 6.4		12.3 ± 6.5	
Estrogen receptor		0.043		0.019		0.023
Negative (4)	11.8 ± 5.8		13.3 ± 6.0		14.8 ± 5.9	
Positive (28)	5.7 ± 4.7		6.0 ± 5.0		3.2 ± 9.1	
Progesterone receptor		0.177		0.169		0.312
Negative (5)	8.4 ± 5.2		9.3 ± 5.8		9.2 ± 11.1	
Positive (27)	6.1 ± 5.2		6.4 ± 5.5		3.8 ± 9.2	
HER-2		0.107		0.177		0.761
Negative (24)	6.1 ± 5.8		6.5 ± 6.2		4.2 ± 9.5	
Positive (8)	7.3 ± 2.6		7.9 ± 3.3		5.8 ± 10.1	
Ki-67		0.003		0.001		<0.001
<22% (16)	3.8 ± 2.5		3.8 ± 2.7		−1.1 ± 8.5	
>22% (16)	9.1 ± 5.8		10.0 ± 6.1		10.3 ± 6.7	
p53		0.219		0.199		0.367
Negative (19)	5.6 ± 5.2		5.9 ± 5.4		3.3 ± 10.2	
Positive (13)	7.7 ± 5.1		8.3 ± 5.8		6.5 ± 8.4	

**Table 3:** Correlations between metabolic parameters and histopathologic variables.

	Mean SUV1	*p* Value	Mean SUV2	*p* Value	Mean RI	*p* Value
Ki-67 LI	*r* = 0.706	<0.001	*r* = 0.744	<0.001	*r* = 0.629	<0.001
Tumor size	*r* = 0.611	<0.001	*r* = 0.592	<0.001	*r* = 0.323	0.07
Number of metastatic Lymph nodes	*r* = 0.167	0.36	*r* = 0.182	0.32	*r* = 0.098	0.59
